# Effect of Various Sugary Beverages on Salivary pH, Flow Rate, and Oral Clearance Rate amongst Adults

**DOI:** 10.1155/2016/5027283

**Published:** 2016-03-08

**Authors:** Rinki Hans, Susan Thomas, Bharat Garla, Rushabh J. Dagli, Manoj Kumar Hans

**Affiliations:** ^1^Department of Public Health Dentistry, K.D. Dental College & Hospital, Mathura, India; ^2^Department of Public Health Dentistry, GDC Kottayam, Kerala, India; ^3^Department of Public Health Dentistry, Best Dental College, Madurai, India; ^4^Department of Public Health Dentistry, Vyas Dental College and Hospital, Jodhpur, India; ^5^Department of Conservative Dentistry & Endodontics, K.D. Dental College & Hospital, Nh 2, Delhi Mathura Road, Chattikara, Mathura 281006, India

## Abstract

*Introduction*. Diet is a major aetiological factor for dental caries and enamel erosion. This study was undertaken with the aim of assessing the effect of selected locally available beverages on salivary pH, flow rate, and oral clearance rate amongst adults.* Materials and Method*. This clinical trial comprised 120 subjects. Test beverages undertaken were pepsi, fruit drink, coffee, and sweetened milk. Statistical analysis was carried out using SPSS version 17. Descriptive statistics, one-way ANOVA, and post hoc Tukey's test were applied in the statistical tests.* Results*. It was found that salivary pH decreased for all the beverages immediately after consumption and the salivary flow rate increased after their consumption. The oral clearance rate of sweetened milk was found to be the least at 6.5 minutes and that of pepsi was found to be 13 minutes. However, the oral clearance rates of fruit drink and coffee were found to be equal at 15 minutes.* Conclusion*. Although it was found out that liquids cleared rapidly from the oral cavity, they had a significant cariogenic and erosive potential. Hence, it is always advised to minimise the consumption of beverages, especially amongst children and young adults to maintain a good oral health.

## 1. Introduction

According to WHO 2012 [1], oral health is defined as “being free of chronic mouth and facial pain, oral and throat cancer, oral sores, birth defects such as cleft lip and palate, periodontal (gum) disease, tooth decay and tooth loss, and other diseases and disorders that affect the mouth and oral cavity.”

Oral health has often been viewed in isolation from the general health. In the past, dental health professionals have focused largely on local reparative treatment of oral disease. However, modern-day dentistry places increased emphasis on the oral disease prevention and recognises the importance of the interrelationship between health of the teeth and oral tissues and the general health of the body [1].

It is well established that a good diet is essential for the development and maintenance of healthy teeth. Diet is a major aetiological factor for dental caries and enamel erosion. Nutritional status impacts the development of the teeth and the host's resistance to many oral conditions, including periodontal diseases and oral cancer [2].

The normal pH of saliva is 6.7 to 7.4 but as bacteria break down the carbohydrates, they release lactic acid, butyric acid, and aspartic acid which bring down the pH of saliva. When the pH level in mouth goes below 5.5 (i.e., the critical pH value), the acids begin to break down the enamel on teeth. The longer the teeth are exposed to a low salivary pH, the more likely the development of dental caries is [3].

Physical state of food plays a very significant role in its cariogenic potential. Liquid sugars, such as those found in beverages and milk drinks, pass through the oral cavity fairly quickly with limited contact time or adherence to tooth surfaces. It is because of their characteristic readiness to flow, little or no tendency to disperse, and relatively high incompressibility. Solid and sticky sugars get stuck to the teeth surface due to their property of adherence. The longer the sugar is stuck to the teeth, the longer the bacteria act on sugars and produce acid thus leading to development of dental caries. Slowly dissolving sources of sugars, such as hard candies, breath mints, and lollipops, have extended exposure time in the oral cavity because the sugars are gradually released during consumption [4].

Over the last few decades, a trend of declining caries in developed countries and an increased caries experience in developing countries has been observed. India too has seen the increasing trend similar to that in the latter group of countries and the trend may continue in the future due to growing globalization [5].

The globalization in turn is bound to increase the availability of processed food items as well as sweets. The association between diet, particularly sucrose, and dental caries has been well-documented in many cross-sectional, longitudinal, and ecological studies. Based on these findings, researchers have advocated limiting the annual sugar consumption to 15 kg per person, both in developing and in developed countries [5].

Though largely preventable, dental caries remains the most prevalent chronic disease in both children and adults. The most susceptible age group is the 17–25 years' age group [5]. In this modern age, use of junk food items and snacking between meals is commonly seen in the younger age group and among their peer groups [5].

Hence, the following study was undertaken with the aim of assessing the effect of selected locally available beverages on salivary pH, flow rate, and oral clearance rate amongst adults.

## 2. Materials and Method

The present study was a clinical trial carried out to evaluate the changes in salivary pH and flow rate after consumption of selected local beverages and to estimate their oral clearance time. The study was carried out in Vyas Dental College and Hospital, Jodhpur, Rajasthan.

### 2.1. Sampling

120 randomly selected undergraduate students from hostel inmates of Vyas Dental College and Hospital, Jodhpur, were examined. These subjects were selected on the basis of the following inclusion and exclusion criteria.

#### 2.1.1. Inclusion Criteria

*They are as follows*:

1. Subjects who were 18–22 years of age.
2. Subjects who were caries-free, that is, with DMFT score = 0.
3. Subjects who were not suffering from any systemic disease or illness.

#### 2.1.2. Exclusion Criteria

*They are as follows*:

1. Subjects who did not give informed consent.
2. Subjects who were using alcohol or tobacco in any form.
3. Subjects who were using any medication at the time of study or in the period of the last 15 days prior to the study.
4. Subjects who were suffering from any systemic illness.

All the study subjects were similar with respect to their age, dietary habits, oral hygiene measures, and other lifestyle factors which could have significant effect on the study results. The time of the day was also standardised for the collection of all the samples.

### 2.2. Ethical Clearance

Before carrying out the present study, the ethical clearance was obtained from the institutional ethical clearance committee.

### 2.3. Informed Consent

Before the start of the study, the purpose and methodology of the study were explained to each of the subjects and informed consent was obtained.

### 2.4. Study Design

The present study was a clinical trial conducted on the hostel inmates of Vyas Dental College and Hospital, Jodhpur. Unstimulated salivary sample was collected for each study subject at least one hour after their breakfast. After the collection of baseline salivary samples (before the consumption of test beverage), the subjects were given one beverage to drink and then stimulated saliva samples were collected at the following fixed time intervals:

- 1st follow-up, immediately after test food consumption.
- 2nd follow-up, 5 minutes after the test food consumption.
- 3rd follow-up, 10 minutes after the test food consumption.
- 4th follow-up, 15 minutes after the test food consumption.

The study subjects were given 4 different beverages to drink for subsequent days and subsequent salivary samples were collected. Carbonated beverage (pepsi), fruit drink (mango drink), and coffee and milk (containing table sugar) were taken under liquids category. Before starting, the intrinsic pH of each beverage was measured. The amount of sugar added in coffee and milk was 1 tablespoon each in 50 mL.

The beverages were consumed as an amount of 50 mL for liquid items.

### 2.5. Collection of Salivary Samples

For the collection of unstimulated saliva, subjects were seated comfortably on a normal chair. The subjects sat with their head bent forward and spat into a sterile calibrated test tube through a sterile funnel. Unstimulated saliva was collected at baseline and at each time interval after test beverage for up to one minute. The salivary pH was directly estimated using the digital pH meter (SIGMA pH meter: model number 131) calibrated with buffers of pH 4 and 7. The accuracy of pH meter was checked at regular intervals to ensure that readings were correct. To measure the pH of the saliva, pH sensitive glass-combination type of electrode was dipped into the collected saliva. The digital reading was allowed to stabilize for a few seconds and the pH reading was recorded. In between the readings, the electrode was cleaned with a stream of distilled water and placed in a standard solution of pH 7. This ensured stable readings and provided a constant check on any drift. The pH of saliva was measured as soon as possible and not later than 10 minutes after the collection of the sample. The flow rate was measured directly from the calibrated test tube after each sample collection. The oral clearance time was estimated on the basis of time taken for the salivary pH to return to the baseline values.

The recording of the data was done by a well-trained recorder who recorded data on a pro forma containing details on the general information and frequency of uptake of the selected test food of each study subject. To minimise bias or errors in the data, an independent observer, blinded to the study's aim, recorded all the digital pH readings.

A *p* value of ≤0.05 was considered significant for all statistical analyses. Statistical analysis was performed using the Statistical Package for Social Sciences (SPSS) software for Windows version 17. The mean and the standard deviation were calculated for the salivary pH and flow rate using descriptive statistics. One-way ANOVA was used to compare the mean salivary pH and flow rate after beverage consumption at different intervals of time. Post hoc turkey's test was conducted to further confirm the findings.

## 3. Results

Table 1 shows the intrinsic pH of various liquid food items. The intrinsic pH of pepsi was found to be the least at 2.04, followed by that of fruit drink (3.89), coffee (6.85), and sweetened milk (7.01). It also shows mean salivary pH at different intervals of time after consumption of different liquid food items. In carbonated beverage (pepsi), the mean salivary pH of the subjects at baseline level was 7.18 ± 0.22. The maximum drop in pH took place at 0 minutes (5.65 ± 0.28). In case of fruit drink, the mean salivary pH at baseline level was 7.05 ± 0.12. The maximum drop in pH took place at 0 minutes (6.08 ± 0.09). In case of coffee, the mean salivary pH at baseline was 7.05 ± 0.12. The maximum drop in pH took place at 0 minutes (6.1 ± 0.09). Although the maximum drop in pH was seen in case of carbonated beverage (pepsi), the oral clearance for all the liquid food items was achieved at 15 minutes.

Table 2 shows the mean salivary flow rate at different intervals of time after consumption of different liquid food items. In case of carbonated beverage (pepsi), the mean salivary flow rate of the subjects at baseline level was 1 ± 0.13. It was observed that the mean salivary flow rate was maximum at 0 minutes (1.78 ± 0.16) which came back to baseline level at 15 minutes (1.02 ± 0.04). In case of fruit drink, the mean salivary flow rate of the subjects at baseline level was 0.83 ± 0.15. It was observed that the mean salivary flow rate was maximum at 0 minutes (1.88 ± 0.49). In case of coffee, the mean salivary flow rate of the subjects at baseline level was 0.8 ± 0.13. It was observed that the mean salivary flow rate was maximum at 0 minutes (1.83 ± 0.49).

Table 3 shows the oral clearance rate of various liquid food items. The oral clearance rate of sweetened milk was found to be the least at 6.5 minutes and that of pepsi was found to be 13 minutes. However, the oral clearance rates of fruit drink and coffee were found to be equal at 15 minutes.

Table 4 shows the comparison of mean salivary pH after beverage consumption at different intervals of time (one-way ANOVA). It was found that the difference in the mean salivary pH at baseline and at different intervals of time after the consumption of pepsi, fruit drink, coffee, and sweetened milk was found to be statistically significant (*p* = 0.000).

Table 5 shows the comparison of mean salivary flow rate after beverage consumption at different intervals of time (one-way ANOVA). It was found that the difference in the mean salivary flow rate at baseline and at different intervals of time after the consumption of pepsi, fruit drink, coffee, and sweetened milk was found to be statistically significant (*p* = 0.000).

## 4. Discussion

### 4.1. Pepsi

The beverage market has in recent years seen drastically increased consumption of aerated drinks [6]. Teenagers and children, whom many fizzy drinks are marketed towards, are among the largest consumers and account for 65% of total sales. Literature reveals that parents' influence, peer pressure, diet fallacies, pleasure, and taste are reasons that lead children to consume these drinks [7].

The changes in drinking patterns also have implications for dental health. A common trend has been observed in the drinking habit of children and adults worldwide. A large number of studies have reported that the quantity of soft drinks consumed was directly proportional to the time spent watching television [8]. The number of sugar-containing snacks and beverages consumed between meals and a late onset of oral hygiene measures correlate positively with plaque accumulation and caries incidence in the primary dentition [9–11]. Soft drinks contain not only sugars but also different organic acids, and they are implicated as an extrinsic cause in the development of dental erosions [12–14].

A prolonged and frequent use of an acidogenic drink, leading to repeated episodes of low plaque pH, would have the potential of demineralisation [15]. However, various host factors like salivary flow rate, buffering capacity, and pH, as well as the concentration of calcium and phosphate in saliva and the frequency of fluid intake, can influence the extent of dental erosion [16].

These drinks are thought to cause damage to the teeth because of two properties: first, the low pH and titrable acidity of some drinks can cause erosion on the enamel surfaces [17, 18] and, secondly, the fermentable carbohydrate in drinks is metabolised by plaque microorganisms to generate organic acids in the dental plaque, resulting in demineralisation and leading to dental caries [19]. The acidogenicity and, hence, the cariogenicity are related to both the extent of acid production and the length of exposure to organic acids [20].

In the present study, the carbonated beverage used was pepsi which contains carbonated water, sugar, caffeine, colouring agents, and acidity regulator as its ingredients. The quantity of sugar added is 10.6 grams/100 grams or 11 teaspoonful of sugar in 300 mL of pepsi. It caused an instant decrease in salivary pH of up to 5.76 ± 1.208. This may be probably due to the fact that the carbonated beverage has increased intrinsic acidic content (maintained in the range of 2-3) and sugar content (14.9 grams in 100 mL) in its composition which are responsible for its high cariogenic and erosive potential. These findings are in agreement with those of other studies as well [14, 21].

An increase of salivary flow rate was observed (2.46 ± 0.993 mL) immediately after the consumption of soft drink. This finding of the study is in agreement with that of the other studies [14, 22, 23].

In the present study, the oral clearance rate of soft drink was found to be 13 minutes. This was probably because the liquids tend to clear faster from the oral cavity. This finding of the study was in agreement with the other studies as well in which the oral clearance rate of beverages was found to be in the range of 10 to 15 minutes [14, 23]. However, contradictory results were quoted in some studies [24, 25] in which the oral clearance rate of the sugar-containing beverages was found to be 30 minutes which was probably as a result of the type of acids and sugars and their concentrations in the beverages used in that study. Liquid sugars pass through the oral cavity fairly quickly with limited contact time or adherence to tooth surfaces because of their characteristic readiness to flow, little or no tendency to disperse, and relatively high incompressibility [26].

### 4.2. Fruit Drink

The fruit drink taken in the study was mango drink. The maximum drop in pH took place at 0 minutes (6.08 ± 0.09). It was observed that the mean salivary flow rate was maximum at 0 minutes (1.88 ± 0.49). The oral clearance rate of fruit drink was found to be 15 minutes. There have been no studies done before on mango drink.

### 4.3. Coffee

Coffee was found to lower the salivary pH but well above the level of critical pH. This might be due to the fact that milk has lactose which has low acidogenicity as found in another study [4]. Nielsen and Popkin reported an initial rise in pH of saliva after milk consumption [7]. Oral clearance of coffee was found to be within 15 minutes which is in agreement with a study which confirmed rapid clearance of liquids from the oral cavity [27].

Beverages are perceived to be quickly cleared from the oral cavity but, on the contrary, beverages sustain a low pH level for a longer duration of time [28, 29]. Similar findings have been reported by Ludwig and Bibby who found that clearance of sugar from the mouth was much more rapid when it was consumed in liquid (beverage) rather than in solid form (snacks) [30].

### 4.4. Sweetened Milk

The reduction in salivary pH of sweetened milk was not significant as the intrinsic pH of milk is very close to the normal (i.e., 7.04) baseline levels. This finding of the study is in agreement with that of other studies [31, 32]. This indicates that milk in its physical state shows an alkaline pH.

The affinity towards milk showed a reflex salivation response [33]. According to Jensen, nasal chemosensory afferents play a significant role in the salivary reflexes [34].

The oral clearance rate of milk was found to be 10 minutes. This may be probably due to the physical state of the milk which enables it to clear from the oral cavity soon. This finding of the study was in agreement with that of the study carried out by Azrak et al. [24] in which the oral clearance rate of milk was found to be 5 to 10 minutes. This result of the present study was in contradiction to the study carried out by Khodadadi et al. [32] in which the oral clearance rate of milk was found to be 30 minutes.

Carbohydrates consumed in liquid form usually do not stay in mouth very long, but if they are consumed often throughout the day, chances for developing dental caries increase. If teeth are constantly exposed to sugary drinks, the acids produced by bacteria remain in oral cavity for a longer time thus causing dental caries and erosion. Drinking sugary beverages with meals will reduce chances for developing dental caries and erosion [27].

## 5. Conclusion

A clinical trial was carried out on 120 subjects to find out the changes in salivary pH, salivary flow rate, and oral clearance rate after consumption of various beverages. These beverages are freely available around the schools and colleges and so are commonly consumed by children and young adults. On examination, it was found out that though liquids cleared rapidly from the oral cavity, they had a significant cariogenic and erosive potential. They caused a major drop in salivary pH just after their consumption. Hence, it is always advised to minimise the consumption of beverages, especially amongst children and young adults, to maintain a good oral health.

## Figures and Tables

**Table 1:** Intrinsic pH and mean salivary pH at different intervals of time after consumption of different liquid food items.

Food items	Intrinsic pH	Baseline	0 min	5 min	10 min	15 min
Pepsi	2.04	7.18 ± 0.22	5.65 ± 0.28	6.21 ± 0.26	6.75 ± 0.16	7.2 ± 0.20
Fruit drink	3.89	7.05 ± 0.12	6.08 ± 0.09	6.4 ± 0.13	6.72 ± 0.17	7.08 ± 0.13
Coffee	6.85	7.05 ± 0.12	6.1 ± 0.09	6.55 ± 0.14	6.82 ± 0.13	7.08 ± 0.09
Sweetened milk	7.01	7.07 ± 0.130	6.71 ± 1.252	6.99 ± 0.393	7.12 ± 0.090	

**Table 2:** Mean salivary flow rate at different intervals of time after consumption of different liquid food items.

Food items	Baseline	0 min	5 min	10 min	15 min
Pepsi	1 ± 0.13	1.78 ± 0.16	1.53 ± 0.16	1.26 ± 0.18	1.02 ± 0.04
Fruit drink	0.83 ± 0.15	1.88 ± 0.49	1.38 ± 0.28	1.15 ± 0.19	0.9 ± 0.17
Coffee	0.8 ± 0.13	1.83 ± 0.49	1.53 ± 0.24	1.15 ± 0.23	0.93 ± 0.10
Sweetened milk	1.23 ± 0.237	2.22 ± 1.07	1.60 ± 0.491	1.42 ± 0.305	

**Table 3:** Oral clearance rate of different liquid food items.

S. number	Liquid food item	Oral clearance rate (in minutes)
1	Pepsi	13
2	Fruit drink	15
3	Coffee	15
4	Sweetened milk	6.5

**Table 4:** Comparison of mean salivary pH after beverage consumption at different intervals of time (one-way ANOVA).

Food item	*F*-value	*p* value	Significant/nonsignificant
Pepsi	18.019	0.000	Significant
Fruit drink	61.546	0.000	Significant
Coffee	71.779	0.000	Significant
Sweetened milk	61.546	0.000	Significant

**Table 5:** Comparison of mean salivary flow rate after beverage consumption at different intervals of time (one-way ANOVA).

Food item	*F*-value	*p* value	Significant/nonsignificant
Pepsi	19.106	0.000	Significant
Fruit drink	13.004	0.000	Significant
Coffee	14.360	0.000	Significant
Sweetened milk	36.138	0.000	Significant

## References

[1] World Health Organization (2012). Oral health. *Fact Sheet*.

[2] Moynihan P. (2005). The interrelationship between diet and oral health. *Proceedings of the Nutrition Society*.

[3] Takahashi N. (2005). *Microbial Ecosystem in the Oral Cavity: Metabolic Diversity in an Ecological Niche and Its Relationship with Oral Diseases*.

[4] Harris N. O., Christen A. G. (1995). Sugar and other sweeteners. *Primary Preventive Dentistry*.

[5] Demircia M., Tuncera S., Yuceokurb A. A. (2010). Prevalence of caries on individual tooth surfaces and its distribution by age and gender in university clinic patients. *European Journal of Dentistry*.

[6] Vartanian L. R., Schwartz M. B., Brownell K. D. (2007). Effects of soft drink consumption on nutrition and health: a systematic review and meta-analysis. *American Journal of Public Health*.

[7] Nielsen S. J., Popkin B. M. (2004). Changes in beverage intake between 1977 and 2001. *American Journal of Preventive Medicine*.

[8] Verzeletti C., Maes L., Santinello M., Vereecken C. A. (2010). Soft drink consumption in adolescence: associations with food-related lifestyles and family rules in Belgium Flanders and the Veneto Region of Italy. *European Journal of Public Health*.

[9] Mattos-Graner R. O., Zelante F., Line R. C. S. R., Mayer M. P. A. (1998). Association between caries prevalence and clinical, microbiological and dietary variables in 1.0 to 2.5-year-old Brazilian children. *Caries Research*.

[10] Habibian M., Roberts G., Lawson M., Stevenson R., Harris S. (2001). Dietary habits and dental health over the first 18 months of life. *Community Dentistry and Oral Epidemiology*.

[11] Jerkovic K., Binnekade J. M., Kruk J. J. V., Most V. D., Talsma A. C., Schans J. J. (2009). Differences in oral health behavior between children from high and children from low SES schools in the Netherlands. *Community Dental Health*.

[12] Rugg-Gunn A. J., Maguire A., Gordon P. H., McCabe J. F., Stephenson G. (1998). Comparison of erosion of dental enamel by four drinks using an intra-oral applicance. *Caries Research*.

[13] Dlaiganm Y. H., Shaw L., Smith A. (2001). Tooth surface loss: dental erosion in a group of British 14-year-old school children part II: influence of dietary intake. *British Dental Journal*.

[14] Moazzez R., Smith B. G. N., Bartlett D. W. (2000). Oral pH and drinking habit during ingestion of a carbonated drink in a group of adolescents with dental erosion. *Journal of Dentistry*.

[15] Tahmassebi J. F., Duggal M. S. (1996). Comparison of the plaque pH response to an acidogenic challenge in children and adults. *Caries Research*.

[16] Millward A., Shaw L., Harrington E., Smith A. J. (1997). Continuous monitoring of salivary flow rate and ph at the surface of the dentition following consumption of acidic beverages. *Caries Research*.

[17] Dlaigan Y. H., Shaw L., Smith A. (2001). Tooth surface loss: dental erosion in a group of British 14-year-old school children, part II: influence of dietary intake. *British Dental Journal*.

[18] Mahoney E. A., Kilpatrick N. M. (2003). Dental erosion: part 1. Aetiology and prevalence of dental erosion. *New Zealand Dental Journal*.

[19] Grobler S. R., Jenkins G. N. (1985). The effects of the composition and method of drinking of soft drinks on plaque pH. *British Dental Journal*.

[20] Mahajan N., Kotwal B., Sachdev V., Rewal N., Gupta R., Goyal S. (2014). Effect of commonly consumed sugar containing and sugar free fruit drinks on the hydrogen ion modulation of human dental plaque. *Journal of Indian Society of Pedodontics and Preventive Dentistry*.

[21] Balappanavar A. Y., Sardana V., Singh M. (2013). Comparison of the effectiveness of 0.5% tea, 2% neem and 0.2% chlorhexidine mouthwashes on oral health: a randomized control trial. *Indian Journal of Dental Research*.

[22] Horswill C. A., Stofan J. R., Horn M. K., Eddy D. E., Murray R. (2006). Effect of exercise and fluid consumption on salivary flow and pH. *International Journal of Sports Medicine*.

[23] Sánchez G. A., Fernandez De Preliasco M. V. (2003). Salivary pH changes during soft drinks consumption in children. *International Journal of Paediatric Dentistry*.

[24] Azrak B., Callaway A., Knözinger S., Willershausen B. (2003). Reduction of the pH-values of whole saliva after the intake of apple juice containing beverages in children and adults. *Oral Health & Preventive Dentistry*.

[25] Mojaver Y. N., Javdi N., Manshaee K. (2008). Influence of soft drink on salivary pH. *The Chinese Journal of Dental Research*.

[26] Demircia M., Tuncera S., Yuceokurb A. A. (2010). Prevalence of caries on individual tooth surfaces and its distribution by age and gender in university clinic patients. *European Journal of Dentistry*.

[27] Decker R. T., Loveren C. V. (2003). Sugars and dental caries. *The American Journal of Clinical Nutrition*.

[28] Holsinger F. C., Bui D. T. (2007). *Anatomy, Function, and Evaluation of the Salivary Glands*.

[29] Moynihan P., Petersen P. E. (2004). Diet, nutrition and the prevention of dental diseases. *Public Health Nutrition*.

[30] Ludwig T. G., Bibby B. G. (1957). Acid production from different carbohydrate foods in plaque and saliva. *Journal of Dental Research*.

[31] Ghosh S., Das S. S., Khan A. (2007). *Rajasthan: Milk Profits from Dairy Farming. Dairy India 2007 Report*.

[32] Khodadadi E., Ghasemi N., Bijani M. P. A. (2013). Total antioxidant property and pH change of dental plaque and saliva in 6–11-year-old children after consumption of flavored milk. *Caspian Journal of Dental Research*.

[33] Pavlov I. P. (1927). *Conditional Reflexes*.

[34] Jensen S. B. (2004). *Flavour Release from Chewing Gum Stimulates Saliva Secretion*.

